# Exposure to cold weather during a mass gathering in the Philippines

**DOI:** 10.2471/BLT.15.158089

**Published:** 2015-09-22

**Authors:** Allison E Gocotano, Fidelita D Dico, Neil R Calungsod, Julie L Hall, Megan L Counahan

**Affiliations:** a World Health Organization Representative Office for the Philippines, Department of Health Compound, Sta Cruz, 1003 Manila, Philippines.; b Department of Health Regional Office VIII, Tacloban, Philippines.; Correspondence to Allison E Gocotano (email: allisongocotano@gmail.com).

## Abstract

**Problem:**

The visit of Pope Francis to the Philippines in January 2015 coincided with a tropical storm. For security reasons, the only road in and out of the area was closed 14.5 hours before the Pope’s arrival. This meant that people had to wait for many hours with little shelter at the site. Medical teams in the field reported high numbers of people with cold stress during the mass gathering.

**Approach:**

To review the event from a public health perspective, we examined the consultations made by medical teams in the field and interviewed key stakeholders, focusing on cold stress as a public health risk.

**Local setting:**

The key reason for the Pope’s visit to Palo and Tacloban was the devastation caused in these cities by typhoon Haiyan in 2013. We estimated that the visit attracted 300 000 people. The medical teams were advised to consider cold stress risks two days before the event but no other measures were taken.

**Relevant changes:**

Of the 1051 people seeking medical care, 231 people were experiencing symptoms of cold stress. People with cold stress ranged from 2 to 89 years of age and were more likely to be female than male, 173 (75%) versus 57 (25%).

**Lessons learnt:**

Planning for mass gatherings should consider a wide range of public health risks, including cold stress. Improved data collection from the field is necessary to maximize the benefits of post-event evaluations and improve public health preparedness. Security measures to ensure the safety of key figures must be balanced with public health risks.

## Résumé

**Problème:**

La visite du pape François aux Philippines en janvier 2015 a coïncidé avec une tempête tropicale. Pour des raisons de sécurité, la seule route permettant d'entrer et de sortir de la zone a été fermée 14.5  heures avant l'arrivée du pape. La foule a donc dû attendre pendant plusieurs heures sur le site qui n'offrait que très peu d'abris. Les équipes médicales envoyées sur le terrain ont signalé un nombre élevé d'individus ayant subi un stress dû au froid lors de ce rassemblement de masse.

**Approche:**

Pour analyser cet événement sous l'angle de la santé publique, nous avons examiné les consultations réalisées sur le terrain par les équipes médicales et interrogé les principaux acteurs, concernant notamment le stress dû au froid en tant que risque pour la santé publique.

**Environnement local:**

Le principal motif de la visite du pape à Palo et Tacloban était la dévastation provoquée dans ces deux villes par le typhon Haiyan en 2013. D'après nos estimations, cette visite a attiré 300 000 personnes. Les équipes médicales ont reçu l'instruction de tenir compte des risques de stress dû au froid deux jours avant l'événement, mais aucune autre mesure n'a été prise.

**Changements significatifs:**

Sur les 1050 individus ayant nécessité des soins médicaux, 231 présentaient des symptômes de stress dû au froid. Les personnes concernées étaient âgées de 2 à 89 ans, les femmes ayant été plus touchées que les hommes – 173 (75%) contre 57 (25%).

**Leçons tirées:**

Il est indispensable de tenir compte d'un grand nombre de risques pour la santé publique, et notamment du stress dû au froid, lors de la planification des rassemblements de masse. Il est par ailleurs nécessaire de réaliser une collecte de données de meilleure qualité sur le terrain pour maximiser les avantages des évaluations post-événement et améliorer la préparation en matière de santé publique. Il convient enfin d'établir un équilibre entre les mesures visant à assurer la sécurité des personnalités éminentes et les risques pour la santé publique.

## Resumen

**Situación:**

La visita del Papa Francisco a Filipinas en enero de 2015 coincidió con una tormenta tropical. Por razones de seguridad, se cerró la única calle para desplazarse dentro y fuera de la zona 14.5  horas antes de la llegada del Papa. Esto hizo que la gente tuviera que esperar durante muchas horas en un lugar donde apenas había donde cobijarse. Los equipos médicos del lugar informaron de un alto número de personas que sufrieron estrés causado por el frío durante la concentración multitudinaria.

**Enfoque:**

Para revisar el caso desde una perspectiva de salud pública, se examinaron las consultas de los equipos médicos en el lugar y se entrevistó a las partes interesadas, enfocando el estrés causado por el frío como un riesgo de salud público.

**Marco regional:**

La razón principal de la visita del Papa a Palo y Tacloban era la devastación que el tifón Haiyan había causado en estas ciudades en 2013. Se estima que la visita atrajo a 300.000 personas. Se alertó a los equipos médicos de que consideraran los riesgos del estrés causado por el frío dos días antes del evento, pero no se tomó ninguna otra medida.

**Cambios importantes:**

De las 1.051 personas que necesitaron asistencia médica, 231 estaban experimentando síntomas de estrés causado por el frío. Las personas con estrés causado por el frío variaban de los 2 a los 89 años y era más probable que fueran mujeres a hombres, 173 (75%) frente a 57 (25%).

**Lecciones aprendidas:**

La planificación de concentraciones multitudinarias debería tener en cuenta una amplia gama de riesgos para la salud pública, incluido el estrés provocado por el frío. Es necesaria una mejor recopilación de datos del lugar para maximizar los beneficios de evaluaciones posteriores al evento y mejorar la disposición de la salud pública. Las medidas de seguridad empleadas para garantizar la protección de personalidades importantes deberían estar equilibradas con los riesgos de salud pública.

## ملخص

**المشكلة:**

تزامنت زيارة البابا فرنسيس إلى الفلبين في يناير/كانون الثاني 2015 مع هبوب عاصفة مدارية. ولدواعٍ أمنية، تم إغلاق الطريق الوحيد للدخول أو الخروج من المنطقة قبل وصول البابا بفترة 14.5 **  **ساعة، مما أدى إلى اضطرار الناس إلى الانتظار لعدة ساعات في ذلك الموقع على قلة ما يحميهم من ظروف الطقس. ووردت تقارير من الفرق الطبية التي تواجدت في هذا الميدان بشأن ارتفاع أعداد الأشخاص الذين أصيبوا بالإجهاد الناتج عن البرودة أثناء احتشاد الجموع.

**الأسلوب:**

من أجل مراجعة الحدث من منظور الصحة العامة، فقد بحثنا في الاستشارات المقدمة من الفرق الطبية في الميدان وأجرينا مقابلات مع الجهات المعنية الرئيسة، مع التركيز على الإجهاد الناتج عن البرودة باعتباره من المخاطر التي تهدد الصحة العامة.

**المواقع المحلية:**

كان السبب الرئيسي لزيارة البابا إلى بالو وتاكلوبان هو الدمار الذي لحق بهاتين المدينتين من جراء إعصار هايان الذي ضرب البلاد في عام 2013. وقدرنا عدد الأشخاص الذين قدموا ابتهاجًا بهذه الزيارة بـ 300,000 **  **شخص. وتم توجيه الفرق الطبية إلى النظر في مخاطر الإجهاد الناتج عن البرودة قبل وقوع ذلك الحدث بيومين، ولكن لم يتم اتخاذ أي إجراءات أخرى.

**التغيرات ذات الصلة:**

كان 231 **  **شخصًا يعانون من أعراض الإجهاد الناتج عن البرودة من بين 1051 **  **شخص باحثين عن الرعاية الطبية. وتراوحت أعمار الأشخاص الذين عانوا من الإجهاد الناتج عن البرودة بين عامين إلى 89 **  **عامًا، وكانت أعداد النساء منهم تميل إلى الزيادة عن أعداد الرجال، حيث بلغت أعدادهن 173 (بنسبة 75%) مقابل 57 (بنسبة 25%).

**الدروس المستفادة:**

عند التخطيط استعدادًا للحشود الجماهيرية فإنه يلزم النظر بعين الاعتبار إلى مجموعة واسعة النطاق من المخاطر التي تهدد الصحة العامة، بما في ذلك الإجهاد الناتج عن البرودة. ويمثل جمع البيانات الميدانية بأسلوب محسَّن ضرورة لتحقيق أقصى حد ممكن من الاستفادة من تقييمات ما بعد الحدث ورفع مستوى التأهب لحماية الصحة العامة. ولا بد من الموازنة بين الإجراءات الأمنية التي تضمن توفير الأمن للشخصيات الهامة ومواجهة المخاطر التي تهدد الصحة العامة.

## 摘要

**问题:**

教皇方济各在 2015 年 1 月访问菲律宾时正逢一次热带风暴。出于安全因素，在教皇到来前 14.5  个小时封锁了出入该地区的唯一道路。这意味着人们不得不在几乎无遮蔽的地点等待数个小时。在这次大规模聚集期间，现场的医疗小组报道了大量人群出现寒冷应激反应。

**方法:**

为了从公共健康角度检讨该事件，我们检查了现场医疗小组做出的会诊，并就作为公众健康风险的寒冷应激这一重点采访了主要的利益相关者。

**当地状况:**

教皇访问帕洛和塔克洛班的主要原因是 2013 年台风海燕对这些城市造成的破坏。我们估计此次访问吸引了 300 000 人。在该事件发生前两天，医疗小组曾收到考虑寒冷应激风险的建议，但是无人采取任何其他措施。

**相关变化:**

在就医的 1051 人中，有 231 人出现了寒冷应激症状。出现寒冷应激反应的人群从 2 岁至 89 岁不等，其中女性人数的可能性大于男性，人数比为 173 (75%) 比 57 (25%)。

**经验教训:**

大规模聚集计划应考虑一系列的公众健康风险，包括寒冷应激。为最大程度提升事后评估的效益并加强公众健康准备工作，改进现场数据的收集很有必要。确保关键人物安全的安保措施必须与公众健康风险保持平衡。

## Резюме

**Проблема:**

Визит Папы Франциска на Филиппины в январе 2015 года совпал с тропическим штормом. По соображениям безопасности единственная дорога, по которой осуществлялось сообщение с местом проведения события в обоих направлениях, была перекрыта за 14.5  часов до приезда Папы. Это означало, что людям пришлось ждать на месте практически без укрытия в течение многих часов. Медики сообщали с мест о том, что в условиях массового скопления людей было много пострадавших от холода.

**Подход:**

Чтобы проанализировать это событие с точки зрения здравоохранения, мы изучили консультации медиков на местах и опросили основных партнеров, основное внимание уделяя стрессу от холода как одному из рисков для здоровья людей.

**Местные условия:**

Основной причиной приезда Папы в Пало и Таклобан были разрушения, вызванные в этих городах тайфуном «Хайян» в 2013 году. По нашим оценкам, визит Папы привлек около 300 000 человек. Врачам было рекомендовано учесть риски стресса от холода за двое суток до события, но никаких других мер предпринято не было.

**Осуществленные перемены:**

За медицинской помощью обратился 1051 человек, из них у 231 были симптомы стресса от холода. Возраст пострадавших от холода составлял от 2 до 89 лет, и обращались больше женщины, а не мужчины — 173 (75%) против 57 (25%).

**Выводы:**

Планирование массовых мероприятий должно проводиться с учетом рисков для здоровья, включая стресс от холода. Необходимо улучшить сбор данных с мест, чтобы извлекать максимальную пользу от последующих оценок, а также повысить готовность органов здравоохранения. Меры безопасности, призванные защитить ключевых фигурантов, должны быть сбалансированы с учетом рисков для здоровья людей.

## Introduction

Between January 15 and 19, 2015, Pope Francis visited the Philippines. On January 17 the Pope visited the cities of Palo and Tacloban, which coincided with the landfall of tropical storm Mekkhala. During his visit in Palo and Tacloban, he conducted an open-air service, visited the archbishop’s residence and Palo Cathedral. The visit attracted large crowds and specific planning was done ahead of time. The pre-event planning focused on hyperthermia, crowd safety and control, but had not considered the risk posed by cold weather. Due to security procedures, the only road in and out of the site was closed to vehicles 14.5 hours before the Pope’s arrival at 18:00 hours, effectively trapping crowds in the area. Before the road closure, attendees were sent by the event organizers to a designated drop-off point 2 km from the site; resulting in many people walking to the site and waiting for the service in the rain for around 19 hours. By 18:00 hours the evening before the visit, all 56 medical teams in the field were prepositioned evenly in the area. The teams used paper logs to record patient consultations. The Philippines Department of Health advised medical teams to consider cold stress risks two days before the event but no additional measures were taken.

## Approach

To assess the number of people seeking care for cold stress during the event, we reviewed the patient logs. Data were analysed separately by the log field “diagnosis” and then by “chief complaint and/or symptom”. The latter was used as a proxy for formal diagnosis. Logs where both fields were blank were excluded. Analysis was conducted using Stata version 12.1 (StataCorp. Lp, College Station, United States of America).

Cold stress was identified when either a medical doctor recorded a diagnosis of cold stress or the log sheet described signs and symptoms consistent with cold stress and no other clinical diagnosis was apparent. Cold stress was diagnosed when axillary temperatures below 36.0 °C were recorded on presentation. Monitoring of vital signs could not be linked to specific log sheets and there were no available data on recovery times.

Twenty-four semi-structured and informal interviews were conducted with key informants to validate findings from log sheets. Respondents included focal points from the Philippines Department of Health, field medical team leaders, referral hospitals, police, the Archdiocese of Palo and attendees.

We estimated the total crowds in Tacloban and Palo at 300 000 people. Pre-event planning had expected one million attendees. Crowds were estimated in four key areas at: (i) the site of the service (designed for 160 000 individuals but officials in attendance suggest crowds were closer to 200 000 people); (ii) the archbishop’s official residence (capacity 3000); (iii) Palo Cathedral (capacity 1500); and (iv) the route between each stop. For the people lining the road, we assumed four people per square metre.^1^ The route taken by the Pope was approximately 11 km long and, based on the estimates from medical teams along the route, approximately 80% of one side of the road was lined with bystanders approximately three metres deep. We thus estimated that 105 600 people were present along the route.

We searched social media (i.e. Facebook and Twitter) for public advisories related to the visit. Almost all suggested that people wear light and comfortable clothing and warned of hyperthermia. Umbrellas and opaque backpacks were prohibited from the site.

## Local setting

Tacloban is situated approximately 360 km south-east of Manila and Palo is a further 12 km south. Typhoon Haiyan devastated these communities in early November 2013 and was the key reason for the Pope’s visit.^2^ Two days before the visit, it was forecast that tropical storm Mekkhala would make landfall about 100 km north-east of Tacloban airport.^3^

The weather station located at the airport monitored ambient temperature, which remained above 22 °C from 03:24 hours on 16 January to 08:19 hours on 17 January. Heavy rain was recorded between 16:00 and 18:00 hours, on 16 January, and was followed by continuous drizzle until 04:00 hours.^4^

## Relevant changes

About half of the medical teams submitted logs (31/56; 55%). These teams were spread evenly throughout the crowd. There were 1051 recorded consultations and the log field “chief complaint and/or symptom” was more often complete than for the field “diagnosis” (1006/1051; 96% versus 526/1051; 50%, respectively). A smaller subset of logs mentioned cold stress even when the “diagnosis” field was blank (38/526; 7%).

Most consultations were at the site of the service and done by staff from the four field hospitals (744; 71%), followed by medical stations (258; 24%) and ambulances (49; 5%) along the route, in the cathedral and at the archbishop’s residence. Cold stress accounted for 22% of consultations (231). No cases were referred for further care and there were no deaths.

People with cold stress ranged from 2 to 89 years of age and were more likely to be female than male (173; 75% versus 57; 25%). The highest proportion was seen among women older than 60 years (Table 1).

**Table 1:** People with cold stress during a mass gathering event in the Philippines, January 2015

Characteristic	Estimated population at mass gathering			No. of cases			Cases per 100 000 population	
	Female	Male		Female	Male		Female	Male
**Age group, years**								
0–19	60 507	63 144		81	34		133.87	53.85
20–59	76 482	77 016		45	17		58.84	22.07
≥ 60	12 282	10 569		39	6		317.54	56.77
Not recorded^a^	–	–		8	0		–	–
**Total**	**149 271**	**150 729**		**173**	**57**		**–**	**–**

^a^ There were nine cold stress cases with unrecorded age (eight females, one unrecorded sex).

**Fig. 1:**
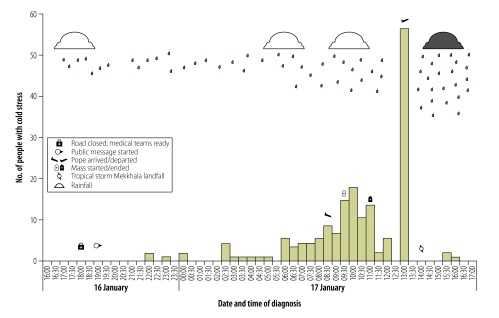
People with cold stress and key events during a mass gathering in the Philippines, January 2015

Note: Three weather bulletins (issued at 05:00, 11:00 and 17:00 hours on 17 January) reported rainfall and wind speed: 7.5 –20 mm/h; 100 kph with gusts to 130 kph respectively.

We learnt from interviews that those present sought medical attention only when they felt unable to continue tolerating the cold and that they had been regularly advised to protect themselves from the weather. Most patients needed assistance to reach the health station and were wet and wearing light clothing on arrival. Blankets and hot drinks were provided on arrival (before recording body temperature). Personal medical histories were not recorded. While the first cold stress case outside the site was reported at 22:00 hours (four hours after the road closure), at the field hospitals where most cases were seen, the first case was at 04:15 hours (almost 12 hours after the road closed). Cases continued to be reported throughout the early morning and peaked after the Pope departed at 13:00 hours (Fig. 1).

Cold stress affected people attending the Pope’s visit in Tacloban and Palo. The main lessons learnt from this event are summarized in Box 1. Most cases were seen in field hospitals at the site of the open air service, which was where people were exposed to the wind and rain for the longest period. While we found the proportion of people with cold stress increased with age, consistent with related studies,^5^^,^^6^ we saw the highest absolute number of cases among young people, which is consistent with the Philippine’s demographic profile (41% of the population are younger than 20 years).^7^ Hypothermia has been associated with poor nutrition and female sex.^5^ Poor nutrition was common in Tacloban and Palo as a result of typhoon Haiyan. About 5.6 million survivors of all ages were at risk of food insecurity.^8^^,^^9^

Box 1 **Summary of main lessons learnt**

• Even in tropical countries, pre-event planning for mass gathering events must consider a wide range of public health risks, including cold stress.

• Improved data collection in the field is necessary to maximize the benefits of a post-event evaluation and improve public health preparedness for mass gathering events.

• Security measures must be balanced with public health risks.

Our study is likely to underestimate the number of people with cold stress. We received information from only half of the medical teams but know the other teams provided medical care to people. The data received were representative of teams at all locations and given the conditions it is likely those not reporting saw people with similar conditions. We were unable to find previous reports of so many people with cold stress associated with a mass gathering in a tropical country. 

## Lessons learnt

This experience has provided important lessons for the Philippines. Tropical cyclones, which commonly result in large-scale evacuations to high density shelters, are permanent hazards. Our study demonstrated cold stress is a legitimate public health concern.

Following the gathering, local health authorities recognized the need to broaden the range of public health risks in pre-event planning. Cold stress was not considered a risk despite the visit occurring towards the end of the cyclone season. The Philippines Department of Health recognized the importance of improving clinical data collection to respond more effectively to a changing situation. Provision of standard individual patient information sheets in addition to summary logs is now being considered for similar events. We recommended procuring supplies (such as thermal blankets) for the clinical management of cold stress cases as a general preparedness measure.

Security measures, such as closing roads and banning umbrellas, increased people’s exposure to the weather. Public health risks need to be considered when planning security measures for mass gatherings.
